# An Integrative Proteomics and Interaction Network-Based Classifier for Prostate Cancer Diagnosis

**DOI:** 10.1371/journal.pone.0063941

**Published:** 2013-05-30

**Authors:** Fu-neng Jiang, Hui-chan He, Yan-qiong Zhang, Deng-Liang Yang, Jie-Hong Huang, Yun-xin Zhu, Ru-jun Mo, Guo Chen, Sheng-bang Yang, Yan-ru Chen, Wei-de Zhong, Wen-Liang Zhou

**Affiliations:** 1 School of Life Sciences, Sun Yat-Sen University, Guangzhou, China; 2 Department of Urology, Guangdong Key Laboratory of Clinical Molecular Medicine and Diagnostics, Guangzhou First People's Hospital, Guangzhou Medical University, Guangzhou China; 3 Institute of Chinese Materia Medica, China Academy of Chinese Medical Sciences, Beijing China; University of Kentucky College of Medicine, United States of America

## Abstract

**Aim:**

Early diagnosis of prostate cancer (PCa), which is a clinically heterogeneous-multifocal disease, is essential to improve the prognosis of patients. However, published PCa diagnostic markers share little overlap and are poorly validated using independent data. Therefore, we here developed an integrative proteomics and interaction network-based classifier by combining the differential protein expression with topological features of human protein interaction networks to enhance the ability of PCa diagnosis.

**Methods and Results:**

By two-dimensional fluorescence difference gel electrophoresis (2D-DIGE) coupled with MS using PCa and adjacent benign tissues of prostate, a total of 60 proteins with the differential expression in PCa tissues were identified as the candidate markers. Then, their networks were analyzed by GeneGO Meta-Core software and three hub proteins (PTEN, SFPQ and HDAC1) were chosen. After that, a PCa diagnostic classifier was constructed by support vector machine (SVM) modeling based on the microarray gene expression data of the genes which encode the hub proteins mentioned above. Validations of diagnostic performance showed that this classifier had high predictive accuracy (85.96∼90.18%) and area under ROC curve (approximating 1.0). Furthermore, the clinical significance of PTEN, SFPQ and HDAC1 proteins in PCa was validated by both ELISA and immunohistochemistry analyses. More interestingly, PTEN protein was identified as an independent prognostic marker for biochemical recurrence-free survival in PCa patients according to the multivariate analysis by Cox Regression.

**Conclusions:**

Our data indicated that the integrative proteomics and interaction network-based classifier which combines the differential protein expression and topological features of human protein interaction network may be a powerful tool for the diagnosis of PCa. We also identified PTEN protein as a novel prognostic marker for biochemical recurrence-free survival in PCa patients.

## Introduction

Prostate cancer (PCa), a clinically heterogeneous-multifocal disease, is the most common malignancy in men and the second leading cause of male cancer-related death [1]. The incidence and mortality from this cause in China appear to be rapidly increasing, and the clinical outcome of PCa patients is difficult to predict. An estimated 20% of PCa patients suffer from recurrent disease after radical prostatectomy or radiation [2]. The 5-year cancer-specific survival rate is close to 80% in men with localized PCa but is only 34% in men with distant metastasis [3]. Prostate-specific antigen (PSA) screening has been extensively used for early detection of clinically localized PCa. However, to date there are no reliable predictors of PCa behavior and aggressive progression. In view of the importance of early diagnosis to the application of curative treatments which are the only hope for increasing the life expectancy of PCa patients, there is an urgent need to develop effective systems which can predict the occurrence of this neoplasm.

Molecular profiling of human cancer has been demonstrated to be a novel approach to investigate this multifaceted disease process. Among various high throughput approaches for molecular profiling, proteome analysis is the most widely based on methods using differential expression on two-dimensional polyacrylamide gel electrophoresis (2D-PAGE) gels or, more recently, two dimensional chromatography followed by mass spectrometry protein identification [4]. It is considered as a powerful tool for global evaluation of protein expression, and has been widely applied in analysis of diseases, especially in fields of cancer research. Two-dimensional difference gel electrophoresis (2D-DIGE) technology, using a mixed-sample internal standard, is now recognized as an accurate method to determine and quantify human proteins, reducing inter-gel variability and simplifying gel analysis [5]. Several groups including our own have adopted this high throughput approach to evaluate the global expression of proteins in several human cancers, including hepatocellular carcinoma (HCC) [6], colorectal cancer [7], esophageal squamous cell carcinoma [8], breast cancer [9], ovarian cancer [10], bladder Cancer [11], PCa [12], [13] and pancreatic cancer [14]. However, there have been the large number of candidate proteins identified using high throughput platforms and it is lack of consistency among different detection systems because of the heterogeneity of the patient cohorts and the difference in platforms. Therefore, it is necessary to identify a reliable and consistent predictor which is robust enough to overcome the variabilities induced by different platforms or different patient cohorts.

Our study group has recently developed a systems biology-based classifier for early diagnosis of HCC by combining differential gene expression and topological characteristics of human protein interaction networks, and also demonstrated that this classifier may efficiently enhance the diagnostic performance for HCC patients [15]. On this basis, in the current study, we intend to develop an integrative proteomics and interaction network-based classifier using the differentially expressed proteins detected by 2D-DIGE in our previous study [12], in order to enhance the ability of PCa diagnosis. We further perform the experimental validation on the clinical significance of candidate PCa markers by Enzyme-linked Immunosorbent Assay (ELISA) and immunohistochemistry analyses.

## Materials and Methods

### Patients and Samples Collection

The study was approved by the Research Ethics Committee of Guangzhou First Municipal People's Hospital, Guangzhou Medical College, Guangzhou, P.R.China. Written informed consent was obtained from all of the patients. All specimens were handled and made anonymous according to the ethical and legal standards.

For 2D-DIGE analysis, four fresh PCa tissues and paired 4 adjacent benign tissues of prostate obtained from 4 PCa patients who underwent transurethral resection of the prostate or radical prostatectomy were provided by Guangzhou First Municipal People's Hospital, Guangzhou, China. None of the patients recruited in this study had adjuvant or neoadjuvant hormonal or radiation treatment before the surgery. The clinicopathological data of the tumor samples are summarized in Table 1.

**Table 1:** Clinicopathological Data of Prostate Cancer Patients Included in 2D-DIGE Experiment.

Patient No.	Age (years)	PSA (ng/ml)	F-PSA (ng/ml)	Gleason Score	TNM Stage
1	54	15.9	1.4	5	T2N0M0
2	77	8.3	1.2	8	T3N0M0
4	70	19.6	1.6	6	T2N0M0
5	80	399.1	50.0	8	T3NxMx

For protein validation by ELISA and immunohistochemistry analyses, 22 cases of prostate cancer tissues and 21 cases of adjacent benign tissues were obtained from patients with PCa who were operated at the Guangzhou First Municipal People's Hospital and Guangdong Provincial People's Hospital, Guangzhou, China. Human PCa tissue microarray (TMA) consisting 112 PCa tissues from Caucasian and African-American PCa patients (aging 46–87 years, mean±SD = 58±7.36 years, TNM staging from I to III) with detailed clinical information were purchased from Jieqing company (Guangzhou, China).The clinicopathological data of these patients are summarized in Table 2.

**Table 2:** Clinicopathological Data of Prostate Cancer Patients Included in ELISA and IHC Assay.

Sample Type & Clinical Features	Experiment Type (cases)	
	ELISA	IHC
**Prostate Cancer**	22	112
**Mean age (range, years)**	75±4.98(62–85)	58±7.36(46–87)
**<60**	0	66
**≥60**	22	46
**Serum PSA Levels (ng/ml)**		
**<4**	2	18
**≥4**	20	91
**Gleason Score**		
**<8**	16	80
**≥8**	8	21
**Clinical Stage**		
**<T2A**	19	60
**≥T2A**	6	47
**Pathological Stage**		
**T2A-T2C**	17	59
**T3A-T4**	7	44
**Metastasis**	0	28
**PSA Failure**	-	36
**Adjacent Benign Prostate Tissue**	21	29

### Identification of differential expression profile of proteins in PCa

The differential expression profile of proteins in PCa tissues compared with adjacent benign tissues of prostate was identified by 2D-DIGE according to the protocols of our previous study [12].

### Network analysis

Network analysis was performed to select essential proteins in disease network as the components of PCa classifier according to the protocols of our previous study [15]. The network representation was generated using GeneGO Meta-Core software (Encinitas, CA). The software interconnected all candidate genes according to published literature-based annotations. Only direct connections between the identified genes were considered. Major hubs were defined as those with more than thirty connections and <50% of edges hidden within the network.

### Integrative proteomics and interaction network-based PCa classifier construction

#### Datasets

To demonstrate this novel classifier, three publicly available datasets of gene expression profiles obtained from Gene Expression Omnibus (GEO, http://www.ncbi.nlm.nih.gov/geo/, Release date: Apr 01, 2012, including 29,123 Series, 9,933 Platforms and 719,101 Samples) were used in this study, including Tomlins_prostate [16] (GEO accession number: GSE6099, 51 PCa samples and 23 non-tumor prostate gland samples), Wallace_prostate [17] (GEO accession number: GSE6956, 75 PCa samples and 14 non-tumor prostate gland samples) and Taylor_prostate [18] (GEO accession number: GSE21034, 150 PCa samples and 29 non-tumor prostate gland samples) datasets. These datasets were randomly separated into the training and test datasets for 100 times.

#### Support vector machine classifier

Support vector machine (SVM) [19], which can address the general case of nonlinear and non-separable classification efficiently, was used to construct our Integrative proteomics and interaction network-based PCa classifier. The goal of an SVM is to find a hyperplane that maximizes the width of the margin between the classes and at the same time minimizes the empirical errors [20]. Here, we selected the radial basis function (RBF) as following formula [21]:
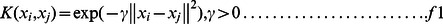
Then, the training dataset was used to input the SVM model so as to calculate the threshold value 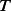 of score by selecting the cutoff value on which the Area under Receiver Operating Characteristic (ROC) Curve (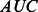) was the biggest. Finally, the SVM classifier decides: if 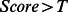, the sample can be predicted as PCa tissues.

### Performance evaluation

The overall performance of PCa classifier was evaluated by two distinct approaches: 5-fold cross-validation test and independent dataset test. The overall predictive accuracy (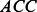) and 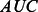 were used to measure the prediction performance of our method. ROC Curve can show the efficacy of one test by presenting both sensitivity and specificity for different cutoff points [22]. Sensitivity and specificity can measure the ability of a test to identify true positives and false ones in a dataset.
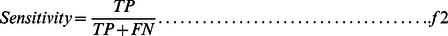


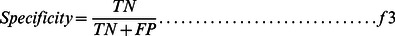


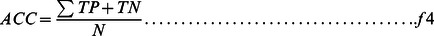
where 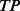, 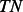, 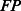, 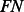 respectively refer to the number of true positive, true negative, false positive and false negative result components in a test, while 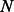 refers to the total number of predicted samples.

The ROC curves are plotted and smoothed by SPSS software with the sensitivity on the 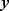 axis and 1-Specificity on the 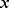 axis.

In the 5-fold cross-validation test, the dataset was randomly divided into 5 sets, four of which were used to train the parameters of the predictive algorithm. The predictive accuracy of the algorithm was then evaluated by the remaining set, and this procedure was repeated five times before sensitivity and specificity against different parameters across five test datasets are calculated for the ROC curve.

### Protein Validation by Enzyme-linked Immunosorbent Assay

The ELISA assay was performed to detect expression levels of potential candidate markers, which were identified as essential proteins by both 2D-DIGE and network analyses according to our previous study [12].

### Protein Validation by immunohistochemistry analysis

The immunohistochemistry analysis was performed to determine the expression patterns and subcellular localizations of potential candidate markers in PCa tissues according to our previous study [23].

### Statistical Analysis

SPSS13.0 software for Windows (SPSS Inc, USA) was used for statistical analysis. Continuous variables were expressed as 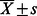
_._ Group comparisons of categorical variables were evaluated using the χ^2^ test or linear by linear association. Comparisons of average means were performed with the independent samples t test or 1-way analysis of variance. The *p* values of less than 0.05 were considered to be statistically significant.

## Results and Discussion

### Identification of candidate PCa markers for network analysis

According to our previous study [12], a total of 60 differentially expressed proteins, including 37 that were up-regulated and 23 that were down-regulated in the PCa tissues, were used for network analysis (the detailed information of this protein list was shown in ).

### Identification of network hub proteins for PCa classifier

**Figure 1:**
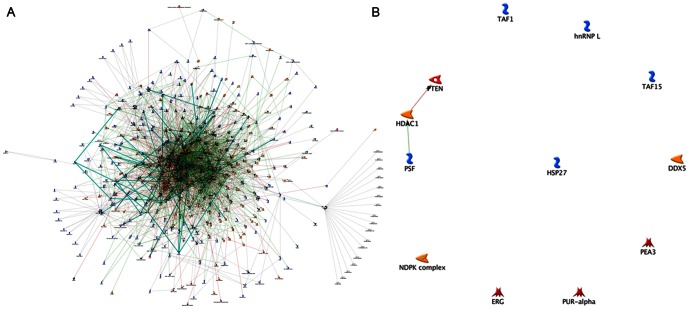
Network for differential expressed genes in PCa tissues (A). **Hub-based network view of 13 differentially expressed hub genes (B).** GeneGO MetaCore was used to generate a network of direct connections among genes selected for analysis. Red, green, and gray arrows indicate negative, positive, and unspecified effects, respectively. Hubs were identified as having more than thirty connections and less than 50% of edges hidden within the network.

To create the network, the proteins (nodes) and published literature-based connections (edges) were plotted using GeneGo-MetaCore. The network architecture is consistent with a scale-free network and represents interactions between individual targets. As the targets with high degrees of connectivity are considered to be the most important components of a network [24], we examined hubs with more than 30 connections and less than 50% of edges hidden within the network. For the network of differential expressed genes in PCa tissues (Figure 1A), 13 hubs were selected to construct their interaction network (Figure 1B): DDX5, ERG, HDAC1, HSP27, NDPK_A, NDPK_B, PEA3, SFPQ (PSF), PTEN, PUR-alpha, TAF1, TAF15, and hnRNP_L (the detailed information of these hub proteins is shown in Table S2). As shown in Figure 1B, three hub proteins (PTEN, HDAC1 and SFPQ) which were interacted with each other closely were chosen to construct our PCa classifier.

### Performance evaluation of PCa classifier

#### PCa classifier construction

On the basis of the gene expression levels of three hubs mentioned above, the PCa classifier was constructed using SVM model. The training dataset was used to training the parameters of PCa classifier and the independent datasets were used to evaluate the performance of this classifier.

#### Independent validation

The independent microarray gene expression datasets were used to test our PCa classifier. Tomlins_prostate [16] (GEO accession number: GSE6099, 51 PCa samples and 23 non-tumor prostate gland samples), Wallace_prostate [17] (GEO accession number: GSE6956, 75 PCa samples and 14 non-tumor prostate gland samples) and Taylor_prostate [18] (GEO accession number: GSE21034, 150 PCa samples and 29 non-tumor prostate gland samples) datasets were randomly separated into the training and test datasets, and this procedure was repeated 100 times. The weights of hub genes and score threshold in the PCa classifier were trained by the training dataset. The predictive accuracy and AUC value of the algorithm was then evaluated by the test datasets, and this procedure was repeated 100 times. Finally, the accuracy and AUC values for different tests were summed to calculate the average and standard error.

The overall predictive accuracy and AUC values of the different PCa classifiers on the Tomlins_prostate, Wallace_prostate and Taylor_prostate test datasets were calculated. As shown in Table 3, the accuracy values of this PCa classifier on different independent test datasets were 85.88∼92.71% and the AUC values were 0.89∼0.93. The AUC value is an indicator of the efficacy of the assessment system. An ideal test with perfect discrimination (100% sensitivity and 100% specificity) has an AUC of 1.0, whereas a non-informative prediction has the area 0.5, indicating that it may be achieved by mere guess. The closer to 1.0 the AUC of a test is, the higher the overall efficacy of the test will be [22]. We found that this PCa classifier had an area approximating 1.0, suggesting that it had a relatively high ability to identify the true PCa tissues against the different independent test datasets.

**Table 3:** Performance of Prostate Cancer Classifiers on Different Independent Test Datasets.

Datasets	Accuracy (%)	AUC
Tomlins_prostate	89.07±5.22	0.90±0.01
Wallace_prostate	85.88±5.79	0.89±0.01
Taylor_prostate	92.71±6.82	0.93±0.03

**Figure 2:**
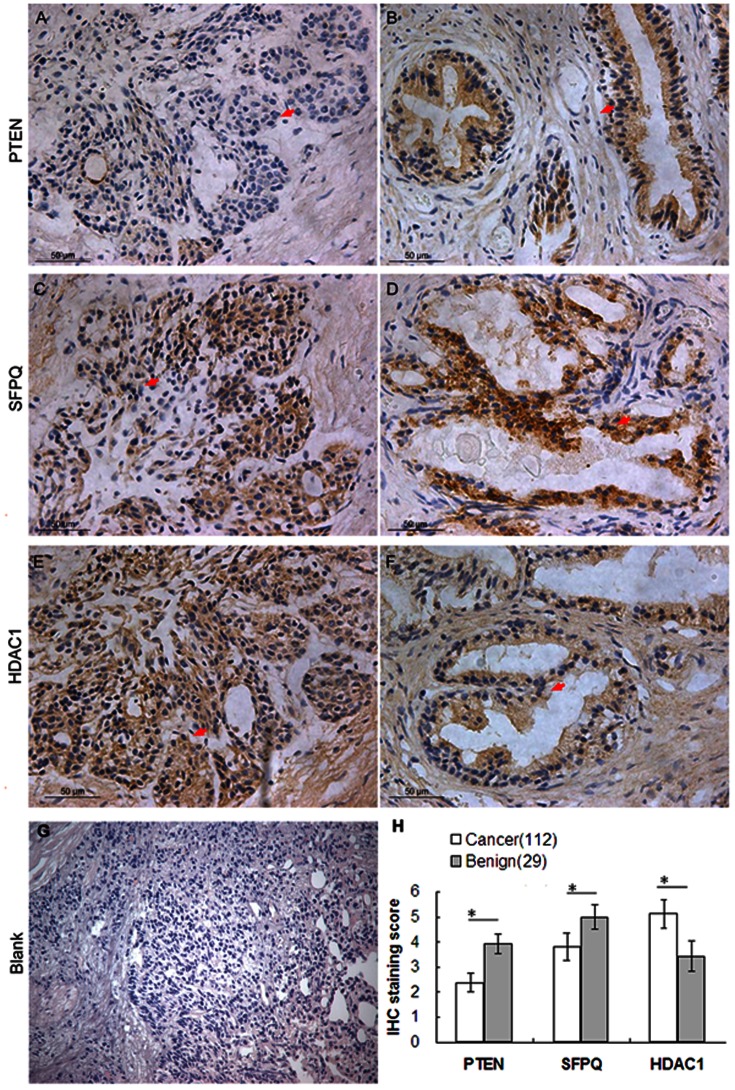
Comparison of the performance of PCa classifier with 13 hubs to that of PCa classifier with 3 hubs. The predictive accuracy and AUC values of the classifier with 3 hubs were both higher than those of the classifier with 13 hubs. But the differences had no statistical significance (all P>0.05).

We selected 3 hubs (PTEN, HDAC1 and SFPQ) from 13 hubs in the network as the component of our PCa classifier, because they were interacted with each other closely. In order to verify the rationality of this selection, we compared the performance of PCa classifier with 13 hubs and that of PCa classifier with 3 hubs. As the results shown in Figure 2, the predictive accuracy and AUC values of the classifier with 3 hubs were both higher than those of the classifier with 13 hubs. But the differences had no statistical significance (all P>0.05), indicating that it may be reasonable to choose the hubs with direct interactions as the component of our PCa classifier.

#### Five-fold cross-validation

We also used the 5-fold cross-validation protocol to evaluate the performance of this PCa classifier. As the AUC is an indicator of the discriminatory power for the classifier, it was used here to evaluate the predictive efficacy of this PCa classifier. As shown in Table 4, the accuracy values of this PCa classifier in all the five tests were 86.32∼92.88% and the AUC values were 0.89∼0.93, suggesting that it has a great reliability and efficacy to identify the true PCa tissues against different test datasets.

**Table 4:** Performance of Prostate Cancer Classifiers for 5-Fold Cross-Validations against the Golden Standard Datasets.

5-fold cross-validations	Accuracy (%)	AUC
5-1	87.41±4.22	0.89±0.01
5-2	86.32±4.06	0.89±0.01
5-3	90.15±4.82	0.91±0.02
5-4	92.88±5.96	0.93±0.03
5-4	89.92±5.33	0.90±0.01

### Clinical significance of PTEN, HDAC1 and SFPQ hub proteins in PCa

**Figure 3:**
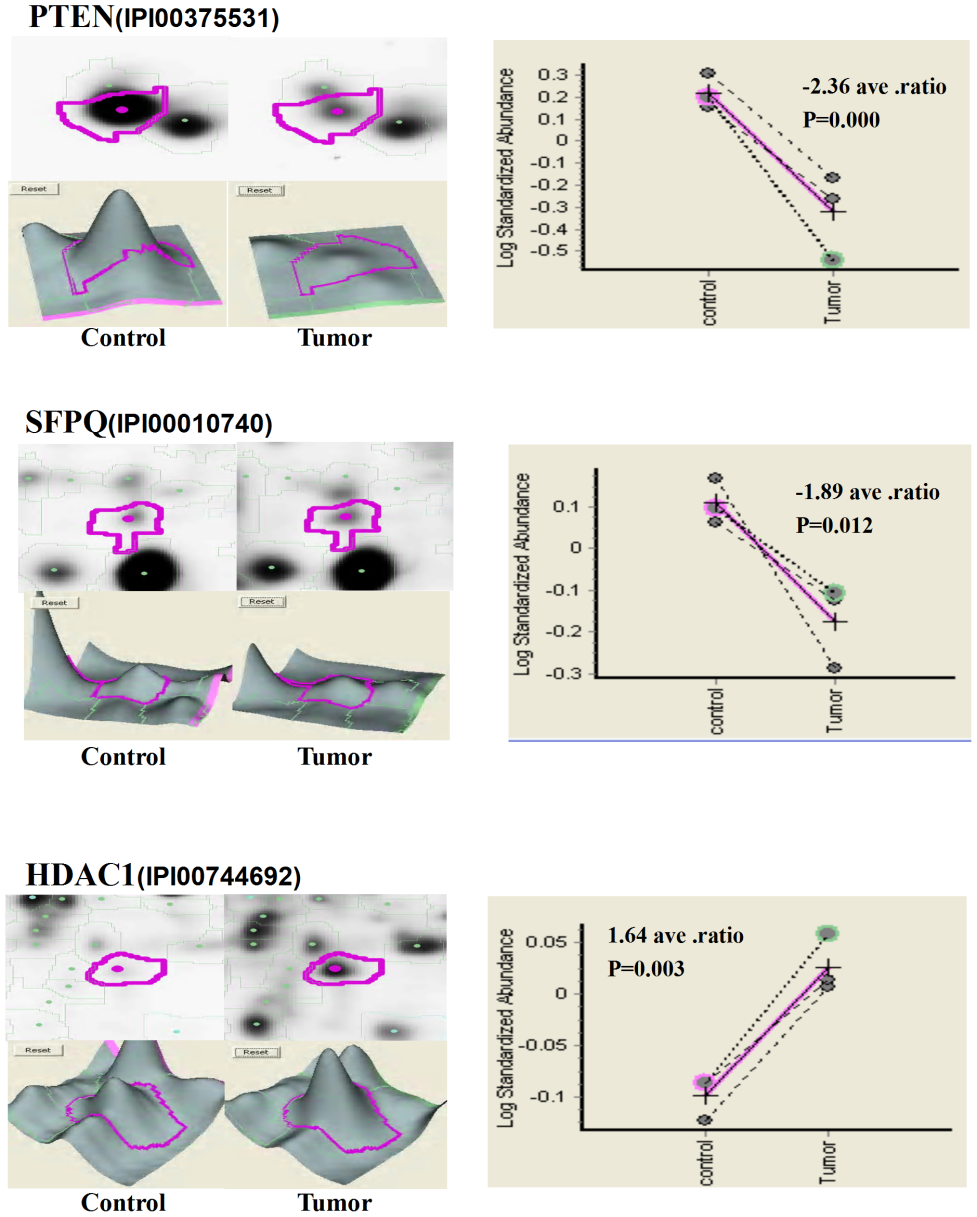
Three-dimensional DIGE images of the 3 hub proteins (PTEN, SFPQ and HDAC1) in prostate cancer tissues and plots of spot intensity values for four paired samples. A paired Student's t-test was applied to all four pairs using DeCyder BVA software.

Nextly, we investigated the associations of three hub proteins: PTEN, HDAC1 and SFPQ, with the clinicopathological characteristics and prognosis of patients with PCa. The 2D-DIGE results of these hubs were shown in Figure 3.

#### PTEN

**Figure 4:**
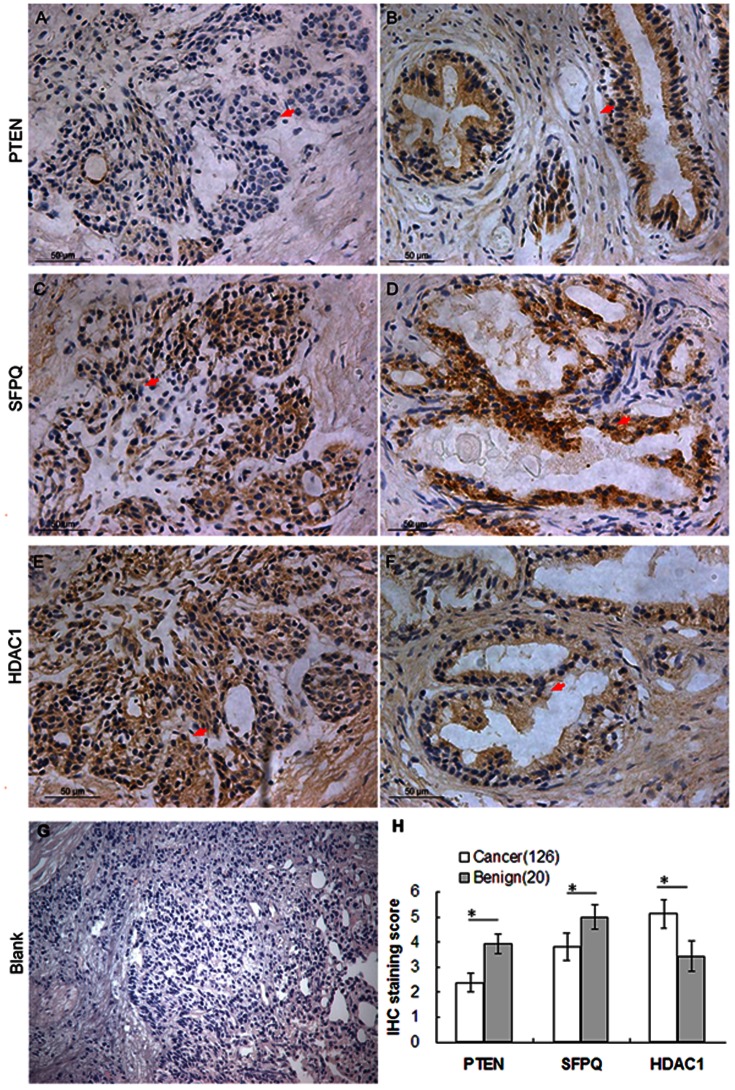
Immunohistochemical staining for PTEN, SFPQ and HDAC1 in PCa and BENIGN tissues (Original magnification×200). A, PTEN weakly positive staining was found in cytoplasm of PCa tissues; B, PTEN strongly positive staining was found in cytoplasm of benign luminal cells; C, SFPQ weakly positive staining was found in cytoplasm of PCa tissues; D, SFPQ strongly positive staining was found in cytoplasm of benign luminal cells; E, HDAC1 strongly positive staining was found in cytoplasm of PCa tissues; F, HDAC1 weakly positive staining was found in cytoplasm of benign luminal cells; G, Negative control for immunohistochemistry analysis; H, Immunohistochemical staining scores of PTEN, SFPQ and HDAC1 in PCa and adjacent benign prostate tissues.

**Figure 5:**
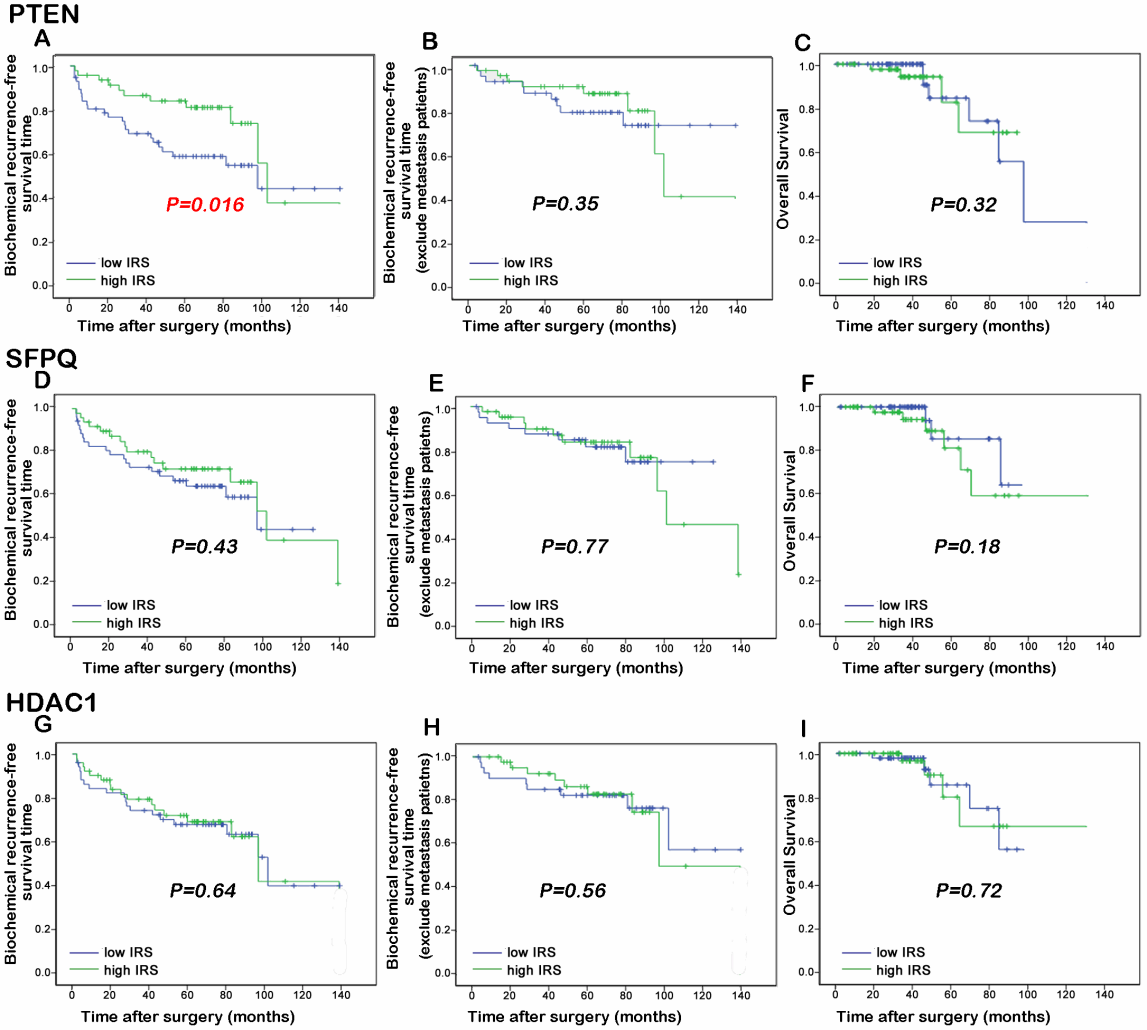
Kaplan-Meier survival curves of the biochemical recurrence-free survival, overall survival and metastasis-free survival for PTEN (A, B and C, respectively), SFPQ (D, E and F, respectively) and HDAC1 (G, H and I, respectively) expression in PCa.

PTEN (phosphatase and tensin homolog on chromosome 10), localized on 10q23.3, is one of the most common tumor suppressor genes in human cancers [25]. It functions as a negative regulator of the PI3K/AKT pathway [26]. Accumulating studies demonstrated the important roles of PTEN in tumorigenesis and tumor progression of PCa. Chaux et al. [27] indicated that loss of PTEN expression may be associated with increased risk of recurrence after prostatectomy for clinically localized PCa; Choucair et al. [28] suggested that PTEN deleted tumors expressing low levels of androgen receptor may represent a worse prognostic subset of PCa establishing a challenge for therapeutic management; Antonarakis et al. [29] found that loss of PTEN expression in primary PCa samples may predict progression-free survival more accurately than clinical factors alone in men with high-risk PCa who receive adjuvant docetaxel after prostatectomy. With the similar results of the previous reports, both ELISA and immunohistochemistry analyses in current study shown that the expression level of PTEN protein in PCa tissues was significantly lower than that in adjacent benign prostate tissues [ELISA assay: 60.96±7.08 (ng/mg) vs. 89.28±20.62 (ng/mg), P<0.001; immunohistochemistry analysis: 2.38±0.37 vs. 3.92±0.40, P = 0.01; Table 5, Figure 4A and B]. In addition, the expression levels of PTEN in PCa tissues with advanced pathological stage and positive metastasis were significantly lower than those with early pathological stage (P = 0.041, Table 6) and negative metastasis (P = 0.006, Table 6). Moreover, the biochemical recurrence-free survival rate of patients with low PTEN expression were significantly lower than those with high PTEN expression (P = 0.016, Figure 5A). Furthermore, the multivariate analyses showed that the down-regulation of PTEN (P = 0.03) was an independent predictor of shorter biochemical recurrence free-survival (Table 7).

**Table 5:** Expression levels of PTEN, HDAC1 and SFPQ hub proteins in Prostate Cancer (Cancer) and adjacent benign tissues (Benign).

Proteins	ELISA (ng/mg)			IHC scores		
	Cancer	Benign	*P*-value	Cancer	Benign	*P*-value
PTEN	60.96±7.08	89.28±20.62	<0.001	2.38±0.37	3.92±0.40	0.01
SFPQ	1.95±2.06	3.75±2.18	0.02	3.81±0.54	5.01±0.48	0.02
HDAC1	6.70±5.02	4.84±3.68	0.03	5.13±0.56	3.44±0.61	0.01

**Table 6:** Associations between Immunoreactivity Scores of PTEN, SFPQ, HDAC1 Proteins and Clinicopathological Features of Prostate Cancer.

Clinical features	Case NO.	PTEN		SFPQ		HDAC1	
		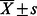	*P*-value	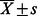	*P*-value	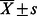	*P*-value
**Age (years)**							
<60	66	2.42±0.31	0.38	3.76±0.39	0.52	5.16±0.60	0.21
≥60	46	2.33±0.38		3.88±0.59		5.08±0.50	
**Serum PSA Levels (ng/ml)**							
<4	18	2.31±0.37	0.58	3.61±0.50	0.59	5.09±0.63	0.46
≥4	91	2.39±0.35		3.84±0.47		5.11±0.55	
**Gleason Score**							
<8	80	2.29±0.29	0.08	3.95±0.36	0.10	5.25±0.55	0.45
≥8	21	2.71±0.32		3.28±0.67		4.68±0.53	
**Clinical Stage**							
<T2A	60	2.56±0.27	0.61	4.44±0.37	0.007**	4.71±0.58	0.015*
≥T2A	47	2.21±0.36		3.01±0.54		5.69±0.54	
**Pathological Stage**							
T2A-T2C	59	2.85±0.31	0.041*	3.94±0.43	0.27	4.96±0.50	0.62
T3A-T4	44	1.79±0.33		3.66±0.55		5.37±0.57	
**Metastasis**							
No	84	2.68±0.29	0.006**	3.95±0.42	0.48	5.04±0.58	0.94
Yes	28	1.47±0.59		3.40±0.65		5.41±0.61	
**PSA Failure**							
**Negative**	66	2.57±0.28	0.13	3.96±0.42	0.35	4.88±0.49	0.20
**Positive**	36	2.05±0.52		3.57±0.58		5.63±0.61	

* <0.05,

** <0.01.

**Table 7:** Biochemical Recurrence-Free Survival in Univariate and Multivariate Analysis by Cox Regression.

	Hazard ratio(95%CI)	*P*-value
**Univariate**		
PTEN	0.40(0.19–0.87)	0.020*
Gleason score	2.75(1.97–3.84)	<0.001**
Preoperative PSA	1.004(1.000–1.007)	0.030*
Pathological tumor stage	4.63(2.26–9.46)	<0.001**
Age	1.013(0.96–1.063)	0.59
Clinical stage group	1.042(0.53–2.037)	0.90
**Multivariate**		
PTEN	1.32(0.40–4.29)	0.03*
Gleason score	2.46(1.61–3.77)	<0.001**
Preoperative PSA	1.006(1.002–1.011)	0.006**
Pathological tumor stage	2.93(1.18–7.26)	0.020*
Age	0.97(.92–1.02)	0.36
Clinical stage group	0.63(.29–1.38)	0.25

* <0.05,

** <0.01.

#### SFPQ

SFPQ (Splicing factor proline/glutamine-rich, also known as PSF) functions as a polypyrimidine tract-binding protein-associated splicing factor that has two coiled-coil domains [30]. It can bind DNA and RNA and is an essential factor for RNA splicing. Xu et al. [31] demonstrated that SFPQ may induce resistance of HeLa cells to 2′,2′- diflurodeoxycytidine as well as other pyrimidine nucleoside analogs; Tanaka et al. [32] reported an SFPQ/PSF-TFE3 gene fusion in perivascular epithelioid cell tumor for the first time. To the best of our knowledge, the involvement of SFPQ in PCa has not been elucidated. In the current study, both ELISA and immunohistochemistry analyses shown that the expression level of SFPQ protein in PCa tissues was significantly lower than that in adjacent benign prostate tissues [ELISA assay: 1.95±2.06 (ng/mg) vs. 3.75±2.18 (ng/mg), P = 0.02; immunohistochemistry analysis: 3.81±0.54 vs. 5.01±0.48, P = 0.02; Table 5, Figure 4C and D]. In addition, the reduced expression of SFPQ protein was significantly associated with advanced clinical stage of PCa tissues (P = 0.007, Table 6). However, our data did not find the prognostic relevance of SFPQ in PCa patients (Figure 5D∼F).

#### HDAC1

HDAC1 (Histone deacetylase 1) is a member of the class I of histone deacetylases which also includes HDAC2, -3 and -8 [33]. It plays important roles in cellular senescence, aging of the liver, myelination, adult neurogenesis and carcinogenesis [34]. HDAC1 interacts with retinoblastoma tumor-suppressor protein and this complex is a key element in the control of cell proliferation and differentiation [35]. Together with metastasis-associated protein-2, HDAC1 deacetylates p53 and modulates its effect on cell growth and apoptosis. In PCa, Patra et al. [36] and Halkidou et al. [37] detected the significantly higher HDAC1 expression in prostate cancer than in benign prostate cell lines and tissues, suggesting that HDAC1 may be associated with the carcinogenesis of PCa. Recently, Lei et al. [38] demonstrated that PTEN loss in PCa may cause reduced expression of NKX3.1 which negatively modulates androgen receptor transcription and consequently the androgen receptor-associated signaling events. They also found that NKX3.1 may engage cell cycle and cell death machinery via association with HDAC1. Consistent with these previous studies, our data shown the up-regulation of HDAC1 protein in PCa tissues when compared with adjacent benign prostate tissues [ELISA assay: 6.70±5.02 (ng/mg) vs. 4.84±3.68 (ng/mg), P = 0.03; immunohistochemistry analysis: 5.13±0.56 vs. 3.44±0.61, P = 0.01; Table 5, Figure 4E and F]. Regarding to its clinical significance, we found that the overexpression of HDAC1 was more frequently occurred in PCa tissues with advanced clinical stage (P = 0.01, Table 6). However, our data did not find the prognostic relevance of HDAC1 in PCa patients (Figure 5G∼I).

## Conclusion

The current study developed a novel classifier of PCa diagnosis that is based on integrating the topological features of protein-protein interaction network with differential protein expression profiles under disease conditions. This systematic integration offers us two main advantages: First, it enables us to sufficiently utilize the protein co-expression information provided by the proteomics data, which is believed to be more informative than expression changes of individual proteins for biomarker identification. Second, network analysis is a powerful tool to understand pathological mechanisms of disease. By integrating the topological features of biological network, some information lost in the differential expression analysis is added to our classifier. More interestingly, by experimental validation using a large number of clinical PCa tissue samples, we also identified PTEN protein as a novel prognostic marker for biochemical recurrence-free survival in PCa patients.

## Supporting Information

Table S1 Differentially Expressed Protein List Identified By 2D-DIGE.
(DOC)

Table S2 Hub proteins of the network of differential expressed proteins in PCa.
(DOCX)
