# Supplementary material for: An Integrative Proteomics and Interaction Network-Based Classifier for Prostate Cancer Diagnosis
Source: PLoS One. 2013 May 30;8(5):e63941. doi: 10.1371/journal.pone.0063941 (PMC3667836; doi:10.1371/journal.pone.0063941)
Supplement: Table S1 — Differentially Expressed Protein List Identified By 2D-DIGE. (DOC) [file pone.0063941.s001.doc]

**Table S1 Differentially Expressed Protein List Identified By 2D-DIGE**

| **Protein_name** | **Accession_No** | **Uniprot_ID** | **Protein_MW** | **Protein_PI** | **Regulation** | **P_value** | **Ratio(fold)** | **Gene_Symbol** | **Protein_ID** |
| --- | --- | --- | --- | --- | --- | --- | --- | --- | --- |
| AMINOACYLASE-1. | IPI00940464 | ACY1_HUMAN | 45856 | 5.77 | up | 0.028 | 1.68 | ACY1 | Q03154 |
| ADENINE PHOSPHORIBOSYLTRANSFERASE. | IPI00218693 | APT_HUMAN | 19595.4 | 5.77 | up | 0.023 | 1.56 | APRT | P07741 |
| CDNA FLJ56442, HIGHLY SIMILAR TO ATP-CITRATE SYNTHASE | IPI00939422 | B4DIM0_HUMAN | 119695.3 | 6.95 | up | 0.001 | 1.5 | ACLY | P53396 |
| CDNA FLJ52710, HIGHLY SIMILAR TO ABHYDROLASE DOMAIN-CONTAINING PROTEIN 14B. | IPI00910706 | B4DNR3_HUMAN | 19784.2 | 5.94 | up | 0.001 | 1.68 | ABHD14B | B4DNR3 |
| COMPLEMENT COMPONENT 1 Q SUBCOMPONENT-BINDING PROTEIN, MITOCHONDRIAL. | IPI00014230 | C1QBP_HUMAN | 31342.6 | 4.32 | up | 0.02 | 1.84 | C1QBP | Q07021 |
| CARNITINE O-PALMITOYLTRANSFERASE 2, MITOCHONDRIAL. | IPI00012912 | CPT2_HUMAN | 73730.4 | 7.27 | up | 0.011 | 2.62 | CPT2 | P23786 |
| ATP-DEPENDENT RNA HELICASE DDX1. | IPI00293655 | DDX1_HUMAN | 82379.8 | 6.8 | up | 0.006 | 1.52 | DDX1 | Q92499 |
| Probable ATP-dependent RNA helicase DDX5 | IPI00017617 | DDX5_HUMAN | 69148.08 | 9.06 | up | 0.019 | 3.09 | DDX5 | P17844 |
| ISOFORM 2 OF ENOYL-COA DELTA ISOMERASE 1, MITOCHONDRIAL. | IPI00398758 | ECI1_HUMAN | 30876.3 | 6 | up | 0.034 | 1.83 | DCI | P42126 |
| ELONGATION FACTOR 2. | IPI00186290 | EF2_HUMAN | 95277 | 6.42 | up | 0.043 | 1.95 | EEF2 | P13639 |
| ELONGATION FACTOR TU, MITOCHONDRIAL PRECURSOR. | IPI00027107 | EFTU_HUMAN | 49843.3 | 6.31 | up | 0.002 | 1.98 | TUFM | P49411 |
| ISOFORM 2 OF ELECTRON TRANSFER FLAVOPROTEIN SUBUNIT BETA. | IPI00556451 | ETFB_HUMAN | 37411 | 8.29 | up | 0 | 1.51 | ETFB | P38117 |
| ETS translocation variant 4 | IPI00017382. | ETV4_HUMAN | 53938.23 | 5.31 | up | 0.016 | 2.19 | ETV4 | P43268 |
| FIBRINOGEN BETA CHAIN. | IPI00298497 | FIBB_HUMAN | 55892.3 | 4.14 | up | 0.042 | 3.33 | FGB | P02675 |
| ISOFORM CYTOPLASMIC OF FUMARATE HYDRATASE, MITOCHONDRIAL. | IPI00759715 | FUMH_HUMAN | 50180.7 | 6.99 | up | 0.008 | 2.03 | FH | P07954 |
| Histone deacetylase 1 | IPI00013774 | HDAC1_HUMAN | 55102.97 | 5.31 | up | 0.003 | 1.64 | HDAC1 | Q13547 |
| ISOFORM 3 OF HETEROGENEOUS NUCLEAR RIBONUCLEOPROTEIN D0. | IPI00220684 | HNRPD_HUMAN | 32814.1 | 7.61 | up | 0.031 | 1.53 | HNRNPD | Q14103 |
| HETEROGENEOUS NUCLEAR RIBONUCLEOPROTEIN L. | IPI00027834 | HNRPL_HUMAN | 64092.4 | 8.46 | up | 0.037 | 1.7 | HNRNPL | P14866 |
| HEAT SHOCK PROTEIN BETA-1. | IPI00025512 | HSPB1_HUMAN | 22768.5 | 5.98 | up | 0.024 | 1.69 | HSPB1 | P04792 |
| UNCHARACTERIZED PROTEIN. | IPI00925196 | IMDH2_HUMAN | 51036.2 | 6.46 | up | 0.018 | 1.92 | IMPDH2 | P12268 |
| ISOFORM 1 OF INORGANIC PYROPHOSPHATASE 2, MITOCHONDRIAL. | IPI00301109 | IPYR2_HUMAN | 37896 | 5.97 | up | 0.013 | 2.34 | PPA2 | Q9H2U2 |
| IG KAPPA CHAIN V-III REGION HIC | IPI00889156 | KV313_HUMAN | 25817.9 | 8.7 | up | 0.015 | 1.8 | IGKV3-20 | P18136 |
| ISOFORM 1 OF METHYLCROTONOYL-COA CARBOXYLASE BETA CHAIN, MITOCHONDRIAL. | IPI00784044 | MCCB_HUMAN | 61294.4 | 6.47 | up | 0.023 | 4.29 | MCCC2 | Q9HCC0 |
| ISOFORM 2 OF NUCLEOSIDE DIPHOSPHATE KINASE A. | IPI00375531 | NDKA_HUMAN | 19640.9 | 5.81 | up | 0.001 | 2.36 | NME1 | P15531 |
| Nucleoside diphosphate kinase B | IPI00026260 | NDKB_HUMAN | 17298.04 | 8.52 | up | 0.007 | 1.74 | NME2 | P22392 |
| NADH DEHYDROGENASE [UBIQUINONE] IRON-SULFUR PROTEIN 2, MITOCHONDRIAL. | IPI00025239 | NDUS2_HUMAN | 52511.7 | 6.26 | up | 0.021 | 1.68 | NDUFS2 | O75306 |
| PYRROLINE-5-CARBOXYLATE REDUCTASE 1, MITOCHONDRIAL. | IPI00941557 | P5CR1_HUMAN | 33339.6 | 7.16 | up | 0.047 | 1.63 | PYCR1 | P32322 |
| CYTOCHROME B-C1 COMPLEX SUBUNIT 2, MITOCHONDRIAL. | IPI00305383 | QCR2_HUMAN | 48412.9 | 7.74 | up | 0.007 | 1.56 | UQCRC2 | P22695 |
| ISOFORM 2 OF HISTONE-BINDING PROTEIN RBBP4. | IPI00877934 | RBBP4_HUMAN | 47555 | 4.74 | up | 0.008 | 2.15 | RBBP4 | Q09028 |
| ISOFORM SHORT OF TATA-BINDING PROTEIN-ASSOCIATED FACTOR 2N. | IPI00020194 | RBP56_HUMAN | 61521 | 8.04 | up | 0.001 | 1.52 | TAF15 | Q92804 |
| ISOFORM LONG OF SPLICING FACTOR, PROLINE- AND GLUTAMINE-RICH. | IPI00010740 | SFPQ_HUMAN | 76101.6 | 9.45 | up | 0.012 | -1.89 | SFPQ | P23246 |
| Transcription initiation factor TFIID subunit 1 | IPI00009891 | TAF1_HUMAN | 212677.24 | 4.96 | up | 0.023 | 2.26 | TAF1 | P21675 |
| TRANSALDOLASE. | IPI00744692 | TALDO_HUMAN | 37516.5 | 6.36 | up | 0.041 | 1.71 | TALDO1 | P37837 |
| T-COMPLEX PROTEIN 1 SUBUNIT BETA. | IPI00297779 | TCPB_HUMAN | 57452.1 | 6.02 | up | 0.037 | 1.67 | CCT2 | P78371 |
| T-COMPLEX PROTEIN 1 SUBUNIT GAMMA. | IPI00553185 | TCPG_HUMAN | 60495.3 | 6.1 | up | 0.032 | 1.56 | CCT3 | P49368 |
| ACETYL-COA ACETYLTRANSFERASE, MITOCHONDRIAL. | IPI00030363 | THIL_HUMAN | 45170.6 | 8.16 | up | 0.014 | 1.5 | ACAT1 | P24752 |
| HEAT SHOCK PROTEIN 75 KDA, MITOCHONDRIAL. | IPI00030275 | TRAP1_HUMAN | 80059.7 | 6.13 | up | 0.036 | 2.17 | TRAP1 | Q12931 |
| CDNA FLJ78096, HIGHLY SIMILAR TO HOMO SAPIENS ACTIN, ALPHA, CARDIAC MUSCLE (ACTC), MRNA. | IPI00930343 | A8K3K1_HUMAN | 42019.9 | 5.23 | down | 0.044 | -5.13 | ACTC1 | A8K3K1 |
| ACTIN, ALPHA CARDIAC MUSCLE 1. | IPI00023006 | ACTC_HUMAN | 41991.9 | 5.23 | down | 0.05 | -11.98 | ACTC1 | P68032 |
| ISOFORM 2 OF ALPHA-AMINOADIPIC SEMIALDEHYDE DEHYDROGENASE. | IPI00936002 | AL7A1_HUMAN | 55331.5 | 6.47 | down | 0.036 | -1.93 | ALDH7A1 | P49419 |
| ISOFORM 1 OF SERUM ALBUMIN. | IPI00745872 | ALBU_HUMAN | 69321.5 | 5.67 | down | 0.003 | -1.52 | ALB | P02768 |
| DIHYDROPYRIMIDINASE-RELATED PROTEIN 3 ISOFORM 1. | IPI00029111 | B3SXQ8_HUMAN | 73864.9 | 6.04 | down | 0.02 | -1.99 | DPYSL3 | Q14195 |
| MACROPHAGE-CAPPING PROTEIN. | IPI00027341 | CAPG_HUMAN | 38493.5 | 5.82 | down | 0.022 | -4.16 | CAPG | P40121 |
| Transcriptional regulator ERG | IPI00005012 | ERG_HUMAN | 54608.39 | 7.01 | down | 0.002 | -2.51 | ERG | P11308 |
| UNCHARACTERIZED PROTEIN. | IPI00219067 | GSTM2_HUMAN | 25728 | 6.02 | down | 0.014 | -2.34 | GSTM2 | P28161 |
| GLUTATHIONE S-TRANSFERASE MU 3. | IPI00246975 | GSTM3_HUMAN | 26542.1 | 5.37 | down | 0.012 | -1.89 | GSTM3 | P21266 |
| GLUTATHIONE S-TRANSFERASE P. | IPI00219757 | GSTP1_HUMAN | 23341 | 5.44 | down | 0.004 | -2.14 | GSTP1 | P09211 |
| KERATIN, TYPE I CYTOSKELETAL 15. | IPI00290077 | K1C15_HUMAN | 49167.1 | 4.71 | down | 0.015 | -8.42 | KRT15 | P19012 |
| CREATINE KINASE B-TYPE. | IPI00022977 | KCRB_HUMAN | 42617.3 | 5.35 | down | 0.026 | -1.57 | CKB | P12277 |
| LIPOMA-PREFERRED PARTNER. | IPI00023704 | LPP_HUMAN | 65703.8 | 7.18 | down | 0.037 | -1.64 | LPP | Q93052 |
| MOESIN | IPI00872814 | MOES_HUMAN | 67644.8 | 6.09 | down | 0.032 | -1.51 | MSN | P26038 |
| MYOSIN REGULATORY LIGHT POLYPEPTIDE 9. | IPI00220278 | MYL9_HUMAN | 19814.4 | 4.78 | down | 0.008 | -5.67 | MYL9 | P24844 |
| PHOSPHOGLYCERATE MUTASE 1. | IPI00549725 | PGAM1_HUMAN | 28785.8 | 6.67 | down | 0.003 | -1.55 | PGAM1 | P18669 |
| ISOFORM 1 OF PROSTATIC ACID PHOSPHATASE. | IPI00396434 | PPAP_HUMAN | 44537.5 | 5.65 | down | 0.001 | -6.65 | ACPP | P15309 |
| ISOFORM 2 OF PROSTATIC ACID PHOSPHATASE. | IPI00289983 | PPAP_HUMAN | 48304.7 | 10.21 | down | 0.007 | -2.84 | ACPP | P15309 |
| Phosphatidylinositol 3,4,5-trisphosphate 3-phosphatase and dual-specificity protein phosphatase PTEN | IPI00012587 | PTEN_HUMAN | 47166.29 | 5.94 | down | 0.000 | -2.36 | PTEN | P60484 |
| TRANSCRIPTIONAL ACTIVATOR PROTEIN PUR-ALPHA. | IPI00023591 | PURA_HUMAN | 34889.4 | 6.06 | down | 0.038 | -1.67 | PURA | Q00577 |
| GLYCOGEN PHOSPHORYLASE, BRAIN FORM. | IPI00004358 | PYGB_HUMAN | 96634.5 | 6.41 | down | 0.023 | -2.54 | PYGB | P11216 |
| ISOFORM 1 OF SERPIN B5. | IPI00783625 | SPB5_HUMAN | 42073.4 | 5.72 | down | 0.03 | -3.96 | SERPINB5 | P36952 |
| ISOFORM 1 OF VINCULIN. | IPI00291175 | VINC_HUMAN | 116649.3 | 5.51 | down | 0.009 | -1.63 | VCL | P18206 |
