# Supplementary material for: An Integrative Proteomics and Interaction Network-Based Classifier for Prostate Cancer Diagnosis
Source: PLoS One. 2013 May 30;8(5):e63941. doi: 10.1371/journal.pone.0063941 (PMC3667836; doi:10.1371/journal.pone.0063941)
Supplement: Table S2 — Hub proteins of the network of differential expressed proteins in PCa. (DOCX) [file pone.0063941.s002.docx]

**Table S2. Hub proteins of the network of differential expressed proteins in PCa**

| Protein Uniprot ID | Gene_symbol | Network_object | All_edges | Hidden_edges |
| --- | --- | --- | --- | --- |
| DDX5_HUMAN | DDX5 | DDX5 | 161 | 0 |
| ERG_HUMAN | ERG | ERG | 136 | 0 |
| HDAC1_HUMAN | HDAC1 | [HDAC1](http://portal.genego.com/cgi/regulation/regulation_info.cgi?id=-1584981221) | 107 | 0 |
| HSPB1_HUMAN | HSP27 | HSP27 | 84 | 0 |
| NDKA_HUMAN | NDPK_A | NDPK_A | 69 | 0 |
| NDKB_HUMAN | NDPK_B | NDPK_B | 65 | 0 |
| ETV4_HUMAN | PEA3 | PEA3 | 59 | 0 |
| SFPQ_HUMAN | PSF | PSF | 48 | 0 |
| PTEN_HUMAN | PTEN | PTEN | 41 | 0 |
| PURA_HUMAN | PUR-alpha | PUR-alpha | 33 | 0 |
| TAF1_HUMAN | TAF1 | TAF1 | 33 | 0 |
| RBP56_HUMAN | TAF15 | TAF15 | 33 | 0 |
| HNRPL_HUMAN | hnRNP_L | hnRNP_L | 31 | 0 |
